# Factors affecting the access to health services among waste collectors in Hanoi, Vietnam: A qualitative study

**DOI:** 10.3934/publichealth.2020039

**Published:** 2020-07-03

**Authors:** Pham Tien Nam, Nguyen Hanh Dung, Nguyen Kim Oanh, Ha Thi Thu

**Affiliations:** 1 Department of Social Work, Hanoi University of Public Health, Hanoi, Vietnam; 2 University of Languages and International Studies, Vietnam National University, Hanoi, Vietnam; 3 Faculty of Social Work, Graduate Academy of Social Sciences, Hanoi, Vietnam

**Keywords:** access to health services, barriers, waste collectors, Vietnam

## Abstract

**Background:**

Waste collection is a common practice in Vietnam. In general, the working and living conditions of waste collectors are poor. Thus, they might be exposed to occupational and environmental risk factors, which affect their health or could further exacerbate their health vulnerabilities. Moreover, they have difficulties to access to health services.

**Objective:**

The aim of this study was to understand factors affecting the access to health services among waste collectors in Hanoi, Vietnam.

**Methods:**

The qualitative design was used for this study. A total of 30 in-depth interviews with waste collectors and 3 focused group discussions were conducted in Hanoi, Vietnam in 2017.

**Results:**

Findings showed the participants considered factors that affect the access to health services among waste collectors such as geographical accessibility, the availability of health facilities, the acceptance of the quality of health services, health insurance, and affordability.

**Conclusions:**

Policy makers concerned with public health and social work need to have the suitable policies in order to promote actions on the access to health services among waste collectors.

## 1. Introduction

Waste management is a major challenge for cities in developing countries. It was calculated that approximately 1.3 billion tons of solid waste were generated in cities around the world in 2012. A predictive figure could increase to 2.2 billion tons in 2025 [1]. There is large-scale participation of workers in waste collection in low and middle-income countries [2]. Waste management consists of a wide range of activities including collecting garbage, collecting and sorting recyclable materials and collecting and processing of commercial and industrial waste [3]. The physical activities of waste collectors are heavy and repeated such as lifting, carrying, pulling, and pushing [4]. Therefore, waste collectors copes with occupational risks. The intensity and type of risks depend on where they work (recycling centers, warehouses, on the streets or in garbage dumps), on their working conditions (informal or organized groups), on the nature of the waste (composition, components and decomposition), and the duration of their exposure [5],[6]. A study in Latin America in 2018 pointed out that the most common reported diseases among waste collectors were osteomuscular disorders (78.7%), arboviruses (28.6%), episodic diarrhea (24.9%), hypertension (24.2%), bronchitis (14.3%), intestinal worms (12.6%) and diabetes (10.1%) [7]. Lucia Botti et al. also found that the waste collectors are affected by low ergonomic conditions and highly risk of musculoskeletal disorders [8]. These are the major reasons that bring waste collectors to access health services.

In Vietnam, there have been several studies on the status of musculoskeletal disorders, occupational accidents and related factors among waste collectors [9],[10], but there have been no study of factors affecting the access to health services among waste collectors. To our knowledge, several studies in India, Amhara region, and Northwest Ethiopia [11],[12] have indicated associated factors to musculoskeletal disorders, and occupational injuries among waste collectors, many factors affecting the access to health services among waste collectors—such as geographical accessibility, the availability of health facilities, the acceptance of the quality of the health services, health insurance, and affordability remain unexplored. In this study, we aimed to understand factors which are affecting the access to health services among waste collectors in Hanoi, Vietnam and are not well reported in previous studies.

## 2. Materials and methods

### 2.1. Study design

This was a cross-sectional study. In our study, the qualitative research was used to understand factors affecting the access to health services among waste collectors in Hanoi, Vietnam. Qualitative research was receiving recognition and was increasingly used in health care research with social and cultural dimensions [13]. Qualitative research was an umbrella term covering an array of interpretative techniques which seek to describe, decode, translate and otherwise come to terms with the meaning, not the frequency, of certain more or less naturally occurring phenomena in the social world [14],[15].

### 2.2. Participants

The subject of the qualitative research focused on waste collectors. They were living and working as waste collectors for more 2 months in Bac Tu Liem district, Hanoi, Vietnam. They were in good state of health and agreed to provide information for this study. The details regarding study participants were reported in section 3.1.

### 2.3. Sample size and sampling techniques

Patton (2002) stated that there are no rules for sample size in qualitative inquiry. In other words, sample size depends on the aim of the study and what is possible, given the time and resources available [16]. In our study, a total of 30 waste collectors had in-depth interviews and 19 waste collectors attended the focus group discussions. Three focus group discussions with 6 to 7 waste collectors each were conducted. Snowball sampling was a recruitment technique in which waste collectors in our study were asked to assist researchers in identifying other potential subjects.

### 2.4. Study content and instruments

The study instruments included in-depth interviews and focus group discussion guides to understand factors affecting the access to health services among waste collectors in Hanoi, Vietnam. We developed the study instruments that are based on Tanahashi framework for effective coverage with health services [17]. For in-depth interviews and focus group discussion guides, these were the main questions: How did you assess geographical accessibility to health facilities? Which health facilities were available at your residence and outside residence? Among those you have listed, how did you assess infrastructures, human resources, medical equipment, medicine, and administrative procedures? How did you assess the quality of health services at the health facilities? Did you have health insurance? How did you use the health insurance? What was your ability to pay for medical services? How much could you afford to pay for it? What were the recommendation for better improvement of the access to health services for waste collectors? These questions were supplemented with any relevant sub questions pending the answers received.

### 2.5. Data collection

Waste collectors were invited to participate in the in-depth interviews and focus group discussions. They also introduced other potential subjects to the research team. All participants received a clear explanation of the study objectives before participating. Their participation in the study was totally voluntary. They could refuse to provide information any moment during the interview without any penalty. An audio recording could only be done after having the consent from the interview subjects. If a participant did not agree to an audio recording, the researchers took detailed notes and captured illustrative quotes. During the survey, interview recordings, field notes, and visual materials were collected. The interviewees' personal identities were coded and quoted anonymously. We spent at least one hour for each in-depth interview and one and a half hours for each focus group discussion.

### 2.6. Quality control of the study

Regarding to the quality control of the study, the guidelines of in-depth interviews and focus group discussions were developed, piloted on-site by the research team, and revised/finalized before the official data collection process. The data collection in the field was conducted by 2 research team members who are working for the Hanoi University of Public Health, have the background of public health or social work, and have at least 5 years of experience in qualitative research. As regards focus group discussion, there were 2 research team members joining, in which 1 member guided focus group discussions, and the other noted the information and meeting minute.

All interviews and focus group discussions were recorded through an audio, transcribed and data-processed with software Nvivo 7.0 according to the study themes. We used thematic analysis to achieve the study's objective. Data were also analyzed by 2 research team members and the analysis was carried out in double.

### 2.7. Ethical approval of the study

The study was approved through the Institutional Review Board of Hanoi University of Public Health (HUPH), with Decision No. 017-315/DD-YTCC. All individual participants included in the study gave informed consent.

## 3. Results

### 3.1. General information

The study results show that the majority of participants were Kinh. They came from the neighboring provinces/cities of Hanoi such as Hung Yen, Nam Dinh, Vinh Phuc, Thai Binh province (over 30 persons). Their age varied from 30 to 65 years old, mainly female (9 male, and 40 female providing the information). There was no difference between male and female, or between the immigrants and the local population in terms of education level. This target group had a relatively low level of education. Most of them completed secondary school and did not follow any religion, only a few follow Christianity.

In addition, all participants in the study were married, and all local people doing this work lived in their own homes with other family members such as their spouses and children. For the immigrant residents, they all lived in a boarding house/rented accommodation. Most of them lived with their spouse or co-workers. Individual index between two genders also varied, in which female waste collectors' heights were from 1 m 50 to 1 m 65, weighing from 40 kg to 55 kg. Meanwhile, male waste collectors were about 1 m 60 to 1 m 70 tall, and their average weight was about 60 kg.

### 3.2. Factors affecting the access to health services among waste collectors

Table 1 below show identified factors affecting the access to health services among waste collectors.

**Table 1.** Factors affecting the access to health services among waste collectors.

Theme	Sub-themes	Codes
Factors	Geographical accessibility	Travel distance
		Travel means
		Travel time
	Availability of health facilities	Infrastructure
		Human Resources
		Medical equipment
		Medicine
		Administrative procedures
	Acceptance of the quality of health services	Attitude of medical staffs
		Waiting time to meet directly medical staff
		Results of healthcare services
	Health insurance	Having health insurance or not having health insurance
		Use of health insurance
	Affordability	Ability to pay
		Borrow money from relatives, friends, and neighbors

#### 3.2.1. Geographical accessibility

According to the study participants who were local, they were more advantageous than the migrants in geographic access to health services. When having common a sickness such as flu, runny nose, headache, leg pain, and etc, they could go immediately to health station at commune level, traditional medicine clinic or pharmacies in their residence. One of the participants said: *“I have not gone to any hospitals but only to the health station for checking my leg injury due to an accident. It is called Yen Be Health Station, about 100 metres from my home. There is also a pharmacy nearby”*. However, the local waste collectors rarely had regular check-ups. They came to hospitals at central level such as Bach Mai hospital, St. Paul hospital, and etc only when having severe illnesses, instead of going to the Health Center of Bac Tu Liem at district level. Moreover, most of the hospitals at central level were located relatively far from their residential area.

According to the local waste collectors, their transportation means to the healthcare providers such as health stations at commune level, pharmacies in their residence, and etc were walking or bike because of the short distance and travel time. A participant shared that *“I often ride a bike to the pharmacy or the health station, usually taking up to 5 minutes”*. In case of getting treatment in the Central-level hospitals, they rode a motorbike. For example, a participant noted *“I usually travel by motorbike in about an hour to get to the hospital at central level”*.

As regards the immigrants, their access to health station at commune level or health centers at district level was much more limited. Most of them did not have access to these health facilities since they were not local. Therefore, when they were not severely ill, they preferred to go to private health clinics, pharmacies in Bac Tu Liem district by the personal means of transportation such as bicycle, motorbike, or walking, instead of getting examination and treatment at public health facilities at commune or district level. One of the participants stated: *“I often go to the pharmacy nearby my home for common illnesses such as flu. I will go to 198 hospital or St. Paul hospital or Bach Mai hospital at central level when having more serious illnesses”*.

Similar to the local, the immigrants would also seek support from bigger hospitals at central level. In case, their health conditions got worse and they could not continue working. They went back to their hometown to prepare for diagnosis and treatment such as treatment fee, belongings, and used public transportation to travel to the hospital at central level to reduce travel costs. One of the participants said: *“I will travel from my hometown to the Bach Mai Hospital by bus. They have from 7 to 8 trips every day; it costs only 7,000 VND (equivalent to 0,3 USD) each trip. Otherwise it is only 1,5 km if go to the commune health station in my hometown”*.

#### 3.2.2. Availability of health facilities

As regards the public health facilities in the Bac Tu Liem district, there were 2 health clinics at district level and 13 health stations at commune level. However, all of the study participants did not reach these clinics. Therefore, our study was unable to assess the specific infrastructure, equipment, medication, the capacity of health workers, and the administrative procedures of these health clinics based on waste collectors' experience.

According to the local participants, the infrastructure of the health stations at commune level was gradually improving. An interviewee shared that *“The Health Station of Yen Be ward is now a renovated 2-storey building. The infrastructure is generally better and more convenient”*. However, the immigrants still considered the facilities of the pharmacies and private clinics that were better. As noted from one of the participants: *“I still think the private pharmacies are more convenient, and better equipped. There are plenty of them around here”*.

Besides, there was not much of difference in the assessment of the local and immigrants in terms of the health facilities' infrastructure outside Bac Tu Liem District as well as the Central-level hospitals. As noted in their evaluation, all of the Central-level hospitals have excellent, modern infrastructure, being credible places for diagnosis and treatment. A participant shared *“I took my wife to Bach Mai Hospital once. We had very good impression of the hospital. The hospital has a nice infrastructure and a clean environment*”. Meanwhile, both the local and immigrants did not highly recommend the infrastructure, medicine of the health services providers in Bac Tu Liem district such as health stations, private clinics and pharmacies. The main reason for this was the lack of medical facilities or synchronous investment in medical facilities. The medicine quality was limited and did not fulfill the study participants' demands. One participant said: *“The health stations at commune level are certainly nearer to our home, but the medical staffs are not qualified enough. Most of them also recommend us going to the hospitals of higher level, since they do not have enough facilities there”*. The differences in health providers in Vietnam were described elsewhere [18],[19].

However, in case their health problems affect their labor productivity, whether they are immigrants or local, they would still go to the Central-level hospitals instead of the Bac Tu Liem district's health service providers. According to them, the equipment, medicine and especially the qualifications of the doctors in hospitals at central level were better than those in health stations at commune level, and health centers at district level. One of the participants mentioned: *“The Central-level hospitals are perfect in my opinion. All of the doctors have doctoral degree with high professional skills, which the district-level or communal-level health providers can not match”*. In addition, according to the assessment of the study participants, the ability to solve the health problems of the health providers in their current residence/Bac Tu Liem district such as health stations at commune level, pharmacies, and private clinics were just average. Besides the mentioned limitations, the study also found that there were still a number of advantages. The current administrative procedures were relatively fast with no frills or long waiting time. This became one of the positive impacts on waste collectors' access to health services in the health facilities of their current residence in Bac Tu Liem district. One of the participants stated: *“There is not a much administrative procedure in the health stations at commune level. Most of the doctors are old. They are very friendly and willing to answer our questions. They also handle tasks very quickly”*.

#### 3.2.3. Acceptance of the quality of health services

The majority of the participants in our study suffered from musculoskeletal disorders. Some of them had other diseases such as trachea, dermatitis, and gastrointestinal tract. One participant shared: *“I often have back pain, knee pain and shoulder pain”.* Moreover, the job caused waste collectors tensions, fatigue, and depression. Many of them had insomnia due to their hard work. A participant said: *“I have had insomnia for 5 years due to my heavy workload and stress about my husband's sickness”.* Therefore, waste collectors had the needs of medical care services utilization.

At the health service providers, the staff's attitudes and behavior were at the forefront of a patient's satisfaction. According to waste collectors, they did not face any difficulty or discrimination in communicating with the medical staffs in the health service facilities inside or outside Bac Tu Liem district, whether they are local or immigrants. One of the participants said: *“My treatment at the Vietnam Cuba Hospital was excellent, as they specialize in Otolaryngology. I was very satisfied with the staff's friendly attitude and good service”*.

At the health service providers in Bac Tu Liem district, for the local participants, they were quite satisfied with the health services provided by health stations at commune level, particularly the services of the preventive health care, epidemic prevention, vaccination for children, pregnant women, and etc. One of the participants mentioned: *“I used to go to Minh Khai Health Station for a gynecological examination. The staff was very friendly and attentive; the examination quality was also very good”*.

However, the pharmacies or private clinics were the frequent destinations for the immigrants who work as waste collectors. A participant shared *“The on-request examinations at the private clinics are more cost-effective, less time-consuming with not too many procedures, and we can receive early diagnostic results. Because there are not too many patients at the clinics, and we can have any type of examinations we want”*. At the large hospitals outside Bac Tu Liem District, both immigrant and local waste collectors were satisfied with the service quality, especially the medical staff's attitudes. One of the participants shared: *“The doctors at 108 Central Hospital were very attentive and gentle, they instructed me every step of the examination”*.

#### 3.2.4. Health insurance

Nearly half of the study participants did not have health insurance. Most of them were the immigrants. A participant shared: *“The first reason is that I do not often get sick. Besides, I am not financially sufficient, so I decided not to buy the insurance”*. Another participant said: *“We are forced to buy for every member of their family, so it is still unaffordable”*.

In addition, unlike the local participants, it was difficult for the immigrants to use health insurance. Therefore, most of them accepted to spend money on unregistered treatment in case their health conditions need treatment. A participant mentioned: *“I often use the services not on correct line, so I do not dare using the insurance. Using the insurance on correct line is very good, but for cases like me, waiting for the insurance is very time-consuming and takes a lot of procedures. That is why I still prefer treatment without the insurance”*. As for the local waste collectors, they underestimated health insurance: *“My husband uses the insurance to cover from 2 to 3 million VND (equivalent from 86,34 to 129,51 USD) of medicine cost, except for several costly medicines that were not covered by the insurance.”*. In addition, they thought that there were also many ways to relieve their illnesses without having to go to the hospitals, so the health insurance was not very necessary. A participant noted: *“I choose to use the traditional medicine and found them effective, which cost from 80,000 to 100,000 VND (equivalent from 3,5 to 4,3 USD) per dose”* or as shared from another waste collector: *“I grew the plants and produced the traditional medicines myself to treat the stomachache. I used to go to the hospitals but switched to this method from 2 to 3 years ago. Therefore, I did not need the health insurance”.*

#### 3.2.5. Affordability

Most waste collectors expressed that if they were seriously ill, they would try to earn or borrow several million VND to pay for treatment. However, if the amount was above ten million VND (equivalent to 432 USD), they would not be able to afford the treatment. Therefore, waste collectors were afraid that they would fall sick, especially the cases in which they were the mainstay of their families. A participant shared: *“Despite being very healthy right now, I am still concerned as I am getting older. I am not sure about my health in the future. If I ever have a terminal disease, I might not be able to afford the treatment”*. Shared a similar concern, a participant said: *“I could afford to pay for the treatment of common illnesses. It is more severe, I would not able to afford in cases of terminal disease”.*

In fact, the majority of study participants did not have severe diseases that need treatment. However, in case they have a serious illness, they could not afford treatment and would try to borrow money from their relatives, friends and neighbors (whether they have or do not have health insurance).

## 4. Discussion

The study results show that the majority of waste collectors in our study was Kinh ethnic, originating from neighboring provinces (over 30 people). The working age of the target group was from 30 to 65 years old, mainly female. The results show that the target group's education level was mainly secondary. Some studies in Brazil and in other countries found some similar results that most of the waste collectors were women, with over 30 years old and low level of education. They had few job options other than waste picking due to their high illiteracy and low education level [1],[20]–[23].

In addition, all study participants were married, and the majority of them lived with their spouse. This was similar with previous studies in Vietnam that nearly 90% of the participants were married and living with husband/wife [9],[10]. Moreover, the study shows that the local waste collectors lived in their own house, and the immigrant residents stayed in rental accommodations. Therefore, many of them such as the immigrants lived with their coworkers or relatives, and their children. This is one of the new aspects that has not been mentioned in any previous study in Vietnam. Our study also found that the musculoskeletal diseases accounted for the majority among waste collectors. This was consistent with the findings from previous studies. Musculoskeletal problems are common among waste collectors such as muscles, joints, tendons, ligaments, bones and the localized blood circulation system [4],[24],[25]. In fact, waste management encompasses many activities including collecting, classifying, recycling and selling garbage. There are many risks involved in this process, from the collection site, during transportation, and at the sites of recycling [26]. These risks effect to waste collector's health problem. A noteworthy point of this study is that waste collectors had mental health problems due to life pressure and job risks.

At the time of the in-depth interviews, waste collectors still underestimated the availability of community-based clinics, mainly due to the lack of medical equipment, asynchronous infrastructure and limited quality medicine. However, in reality, the infrastructure, equipment, and medicines at the health center at district level and health stations at commune level were being improved and rebuilt such as the health station of Phuc Dien ward. Despite being unable to timely meet the people's needs of healthcare in the district, this is still regarded as one of the innovative steps in the local healthcare activities. According to the interviewees, the equipment, medicines and especially the professionalism of doctors in hospitals at central level were better than the health stations, pharmacies, private clinics, and etc. in Bac Tu Liem district. This made it even more common for the migrants or local people to go to central hospitals instead of going to healthcare facilities at their current residence/Bac Tu Liem district.

The waste collectors, who were interviewees had no the routine of regular health examination due to their limited budget. This did not allow them to prioritize the disease prevention. They might know that the regular health examination and early detection would lead to less money to spend, shorter time for treatment and higher recovery possibility. A study by Bogale indicated that in low-income countries, waste collectors have low socio-economic status such as poverty, poor housing conditions and poor nutrition that impacts to their regular health examination [4]. Routine checkup program for all waste collectors is mandatory to keep them safe and secure [27]–[29].

Nearly half of the participants did not have health insurance, mostly the immigrants. This differed from the study of Hoang Thi Ngan in 2017 that the majority of the waste collector are formal workers having health insurance and social insurance. These are paid by their company [10]. As informal workers, waste collectors have difficulties to have access to social benefits such as health insurance, pensions and unemployment insurance [30]. Unlike the local people, it is difficult for the migrants to use health insurance. Because each time they want to use health insurance at registered level, they have to go back to their hometown and follow the procedures which are pretty cumbersome in their opinion. Therefore, most of them accepted to spend money for treatment at unregistered level (for cases that need treatment). The local people, on the other hand, underestimated the health insurance. From their viewpoint, the insurance did not cover the needed medications which are pretty expensive. For those who have health insurance, they were still very concerned since the cost of healthcare service covered by health insurance was relatively low. Therefore, the waste collectors must pay extra for some medicines and healthcare services. In particular, the more severe the disease is, the more extra cost they have to spend on medicines. This has an impact on their family's economy while these objects are financially unstable. If they are ill, they are no longer healthy to continue their work, and the economy will be a difficult problem to solve, turning to a burden on not only themselves but also on their families. Previous studies pointed out that it would be more difficult for the workers including waste collectors to access to health services if a disruption occurs in the system of healthcare service [31] such as COVID-19 pandemic. This may be explained that waste collectors are one of vulnerable groups in society and need to supported in emergency situation.

## 5. Limitations

Our study is also subjected to several limitations. First, we used snowball sampling to recruit the participants; therefore, it could be a bias on gender. Second, we did not have an interviews with stakeholders in our study. Third, we did not categorize more details about the different health care services offered by health providers. Lastly, our findings may not generalizable to waste collectors in the whole country of Vietnam. Therefore, there is a need for future studies to address these limitations.

In conclusion, waste collectors were coping with difficulties to access to health services such as geographical accessibility, the availability of health facilities, the acceptance of the quality of health services, health insurance, and affordability. We call for prospective studies to confirm our findings. Moreover, our results may suggest that stakeholders in the field of health and labor, social affairs need to manage the group of waste collectors in community and pay attention to the difference between the local and immigrant in terms of access to healthcare services.
